# Resilience to depression and unchanged quality of life in Sardinian old adults during COVID-19 lockdown

**DOI:** 10.3389/fpsyt.2025.1642413

**Published:** 2025-09-03

**Authors:** Giulia Cossu, Michela Atzeni, Elisa Cantone, Viviana Forte, Massimo Tusconi, Goce Kalcev, Alberto Maleci, Roberta Montisci, Caterina Ferreli, Laura Atzori, Diego Primavera, Serdar M Dursun, Fabrizio Bert, Enzo Tramontano, Mauro Giovanni Carta

**Affiliations:** ^1^ Department of Medical Sciences and Public Health, University of Cagliari, Cagliari, Italy; ^2^ University Hospital of Cagliari, Cagliari, Italy; ^3^ Neurochemical Research Unit, Department of Psychiatry, University of Alberta, Edmonton, AB, Canada; ^4^ Department of Public Health and Pediatrics Sciences, University of Torino, Torino, Italy; ^5^ Department of Life and Environmental Sciences, University of Cagliari, Cagliari, Italy

**Keywords:** resilience, depressive symptoms, lockdown, mental health, aging, older adults, COVID-19

## Abstract

**Introduction:**

Older adults were expected to be particularly vulnerable to depressive symptoms during the COVID-19 lockdown due to social isolation, disruption of daily rhythms, and higher mortality risk. However, some studies have reported unexpected resilience in this population. This study aimed to assess resilience, operationalized as stability in depressive symptoms and health-related quality of life (H-QoL), before and during lockdown among older adults in Sardinia, Italy.

**Methods:**

We conducted a cohort follow-up study including community-dwelling older adults aged ≥65 years, originally enrolled in a randomized controlled trial promoting active aging. Participants were assessed at baseline (March-April 2019) and during lockdown (March-April 2020) using the Patient Health Questionnaire-9 (PHQ-9) and the Short-Form Health Survey (SF-12). Three groups were analyzed: 1. Study cohort, same participants assessed before and during lockdown (pre-post comparison). 2. National matched sample, extracted from a large Italian epidemiological survey, matched by age and sex, for comparison of SF-12 scores. 3. Regional matched sample, extracted from a Sardinian epidemiological survey, matched by age and sex, for comparison of PHQ-9 prevalence rates. Statistical analyses included ANOVA for continuous variables and Chi-square tests for proportions.

**Results:**

Ninety-three older adults (mean age 73.4 ± 5 years; 53.8% female) were included in the study cohort. No significant differences were found in SF-12 scores between baseline and lockdown (35.7 ± 4.5 vs. 34.3 ± 6.4; F (1,84) = 2.999, p = 0.085) or in PHQ-9 scores (2.3 ± 3.0 vs. 2.3 ± 3.5; F (1,84) = 0.035, p = 0.851). The proportion of participants with moderate/severe depressive symptoms (PHQ-9 ≥10) showed a non-significant increase (3.2% vs. 6.5%; p = 0.305). Comparisons with the national and regional matched samples also revealed no statistically significant differences.

**Discussion:**

Older adults in Sardinia demonstrated resilience during lockdown, maintaining stable levels of depressive symptoms and quality of life. Potential protective factors include strong family networks, maintenance of daily rhythms, and limited digital overexposure. These findings align with reports from other Western countries and warrant further research into cultural and social determinants of resilience in aging populations during crises.

**Clinical trial number:**

NCT03858114.

## Introduction

Depressive disorders in older adults increase the disability’s burden and the risk of non-self-sufficiency (1). This is particularly relevant in Europe, where it is estimated that by 2050, 34% of the population will be over 60 years old (2). In comparison, the median age was 42.5 years in 2019, more than twice that of Africa (2). The burden of chronic diseases increases with ageing, resulting in a higher number of disability-adjusted life years (DALYs) which impact with a significant effect on social and healthcare costs (3). The issue of supporting older adults, particularly those with depression, becomes more crucial during severe crises such as natural disasters and in circumstances like the recent pandemic. These situations have highlighted the vulnerability of nursing homes, both for self-sufficient older adults and those requiring assistance due to non-self-sufficiency (4, 5). However, studies conducted in high-income countries have established an unexpected resilience among older adults living at home during the pandemic, reporting lower rates of distress and depressive disorders compared to other age groups (6–9). A greater vulnerability to depression was expected due to restricted access to healthcare, social interactions with family members and stakeholders. Previous research has shown that older adults may exhibit distinct patterns of depressive symptoms and quality of life trajectories when stratified by gender, particularly under conditions of social restriction; especially women often present a higher baseline burden of depressive symptoms, possibly reflecting a combination of biological susceptibility, differential stress appraisal, and social role expectations (10, 11). However, women may also display greater relative improvement in mental health outcomes when engaged in structured, socially oriented programs (12). Moreover previous evidence from active aging interventions, ranging from moderate-intensity physical activity to cognitively and culturally enriching activities, indicates a consistent benefit on mood regulation, perceived well-being, and health-related quality of life (H-QoL), with some studies suggesting a more pronounced effect in female participants (13, 15). Evidence from the Italian context reinforces these findings, showing that both physical and culturally engaging interventions can enhance resilience in older adults, sustain regular social and biological rhythms, and mitigate the impact of stressors such as the COVID-19 lockdown (16). These findings support the inclusion of gender-stratified analyses and the examination of active aging programs as potentially modifiable protective factors in the relationship between depressive symptoms, quality of life, and resilience in older populations. The interruption of usual daily rhythms/routines activities during lockdown, identified as a risk factor for mood disorders (17), may have led to an even greater impact on aged individuals. Also, older adults were expected to have more difficulty adapting to the growing use of remote care and telemedicine during lockdown (18). Additionally, they faced the psychological pressure of knowing that their age exposed them to an elevated risk of mortality due to the Sars-COV2 infection (19). Concerning this, it has been suggested that studies on general or specific populations during lockdown were often performed on non-representative samples. Participation was voluntary, and assessment tools were most frequently administered online. This may have introduced a selection bias, as older participants who took part were more likely to be in good health/well-being and have a higher level of education (20, 21). Our research group already assessed the frequency of depressive symptoms and the perception of quality of life in samples of older adults on an active aging project conducted one year before lockdown (22). The pre-post evaluation suggests that frequency of depressive symptoms (23) and depressive episodes (24) together with H-QoL (25) did not worsen during the lockdown. Having regular biological and social rhythms one year before the lockdown was found as a protective factor against depression during the lockdown (26). However, the relatively small sample size limited us to make solid conclusions.

The aim of the present study is to compare the frequency of depressive symptoms, depressive episodes, and the level of H-QoL in the overall sample of older adults -including both the experimental arm (assigned to physical activity interventions) and the active control arm (assigned to culturally engaging activities)- assessed during the lockdown and one year before it. Also, these outcomes will be compared with identical indicators in larger, matched samples by age and sex, adopted from databases representative of the populations from the same background. This methodology improves the study’s power, allowing for more robust conclusions and the estimation of potential associations between depressive symptoms and socio-demographic variables.

## Materials and methods

### Design and settings

This cohort follow-up study was conducted one year after an active aging intervention and coincided with the COVID-19 lockdown. Participants were assessed at T0 (March-April 2019) and at T1 (March-April 2020), corresponding to the final follow-up one year later, during the lockdown. In the study sample, outcome measures obtained at T0 were compared with those recorded at T1. H-QoL scores from T1 were also compared with those from a sample drawn from the database of an epidemiological survey conducted in six Italian regions before the pandemic (27). Depressive symptoms were compared with a sample obtained from the data bank of an epidemiological survey carried out in Sardinia (28). Both epidemiological surveys employed assessment tools similar to those used in the present survey of older adults.

### Sample and recruitment

The study sample comprised community-dwelling individuals aged 65 years or older, with no restriction by sex, residing within the metropolitan area of Cagliari, Sardinia. Recruitment was conducted over a fixed 3-month enrollment period (March-May 2019) through public notices disseminated via local media, referral by general practitioners (GPs), and outreach by local community associations and senior centers. Older adults affiliated with the University Hospital were also approached, following prior communication with their GPs. Individuals with chronic medical conditions were eligible and represented a substantial proportion of the sample. To be eligible for enrolment, individuals had to be deemed fit to participate in mild-to-moderate intensity physical activity and to obtain a medical clearance certificate confirming their suitability. Exclusion criteria included severe obesity, major functional limitations, or medical conditions that would preclude safe participation. The final analytic sample consisted of 93 eligible and consenting participants. Operational constraints, specifically, the requirement for supervised, in-person sessions delivered in small groups of 8-12 participants, and the geographic limitation to a single metropolitan area- restricted the total number of participants. Nonetheless, the analytical design, for the purposes of the present study, incorporated matched control samples from large, community-based epidemiological surveys, thereby enhancing statistical power and external validity (29).

A brief description of the intervention and research arms is provided solely to describe the activities, not for analytical purposes in the present work, and is fully reported elsewhere (22). The experimental intervention consisted of a 12-month, twice-weekly, 60-minute supervised physical activity program delivered to small groups (8-12 participants). Sessions combined aerobic, strength, balance, and flexibility exercises, with training intensity maintained at 60-80% of the Heart Rate Reserve and continuously monitored. The active control group participated in matched-duration cultural activities, such as museum-based guided tours within the metropolitan area of Cagliari, designed to provide comparable social engagement without a physical training component. Participants were randomized by sex and age strata, with assessments conducted at baseline and after 12 months.

### Tools

This observational cohort follow-up study is part of a broad academic collaboration aimed at investigating healthy aging through a multidisciplinary approach, using a wide range of validated instruments. A detailed description of the full assessment protocol and methodology is provided elsewhere (22).

At baseline (T0), all participants completed a structured sociodemographic and health questionnaire administered in person by trained research staff. Recorded variables included: age, sex, marital status, educational level, living arrangement (alone, with spouse/partner, or with extended family), employment/retirement status, primary source of income, presence of chronic medical conditions (e.g., hypertension, diabetes, cardiovascular disease, respiratory illness), current use of prescription medications, and any previous psychiatric diagnosis or treatment. Depressive symptoms were measured through the Patient Health Questionnaire (PHQ-9) (30), a 9-item tool conceived for screening depressive episodes and assessing and monitoring the severity of depressive symptoms, including all items concerning the Diagnostic and Statistical Manual of Mental Disorders (DSM) (28/30) criteria for a diagnosis of major depressive episode, integrated into a simple self-report instrument (31). The internal consistency is Cronbach’s α = 0.89 (30); the tool was used in its italian version (32, 33). The severity of each symptom/item is measured on a Likert scale ranging from 0 (corresponding to “not at all”) to 4 (“maximum severity or every day”). A cut-off score of 4/5 indicates a depressive episode of at least mild severity, while a cut-off score of 9/10 indicates a depressive episode of at least moderate severity (30). The Short-Form Health Survey (SF-12) (34, 33) was utilized to assess H-QoL based on physical and psychological components. SF-12 is a questionnaire composed of 12 items, constructed to measure how an individual’s health is perceived to impact their daily life;. Higher scores indicate a better perception of health-related quality of life. Its internal consistency is Cronbach’s α = 0.94 (35). For the present study, internal consistency was assessed at baseline (T0) for both tools. The PHQ-9 showed good internal consistency with a Cronbach’s α coefficient of 0.86, in line with previous Italian validation studies (33). The SF-12​ total score exhibited good internal consistency (Cronbach’s α = 0.81), consistent with values​ observed in previous Italian population-based studies (36, 37).

### Statistical analysis

Statistical analysis involved the comparison of pre-post SF-12 mean scores in the study sample (as the dependent variable). Additionally, SF-12 scores from the study sample were compared with those from the data bank of an epidemiological survey carried out in six Italian regions prior to the pandemic (27). The pre-post frequency of the depressive episodes in the study sample, based on PHQ-9 cut-off scores of 4/5 and 9/10, was also assessed, as well in pre-post sub-samples subdivided by sex and age (<75 years versus >74years). Furthermore, the frequency of depressive episodes in the study sample, screened by PHQ-9 with cut-off score of 9/10, was compared with that in the sample from the data bank of an epidemiological survey carried out in Sardinia (28). The two samples from the databases of the two community surveys were extracted after block randomization. For each person in the study sample, two participants, matched by age and sex, were selected to create a randomization cell. In the sample from the Italian study (27), matching was conducted with individuals of the same age, while in the smaller Sardinian study sample (28), matching was performed with individuals having an age difference of less than two years. Once a record was extracted from the database, it was excluded from subsequent randomizations.

Statistical analysis for numerical data was calculated using one-way ANOVA for matched samples (pre-post) or standard ANOVA for non-matched samples (comparison between the study sample and the sample drawn from the Italian community survey databank). Statistical analysis for nominal data was measured by Chi square test or Fisher exact text using the Castellan method for comparing differences by time and group (38). More specifically: statistical analysis involved the comparison of pre-post SF-12 and PHQ-9 mean scores in the study cohort using one-way repeated measures ANOVA for dependent groups, as the same participants were assessed at baseline (T0) and during lockdown (T1). Comparisons between the study sample and the matched control samples from the two epidemiological surveys (27, 28) were conducted using one-way ANOVA for independent groups. All tests were two-tailed. The analyses were not part of a generalized mixed model (GMM) framework. No *post-hoc* corrections for multiple comparisons were applied, as the number of planned comparisons was limited and the analyses were primarily exploratory; however, exact p-values are reported for transparency, allowing readers to interpret statistical significance in context. Nominal data were compared using Chi-square tests or Fisher’s exact tests, applying the Castellan method for stratified analysis when appropriate (39).

### Ethics approval

All participants provided written informed consent. They were informed that their data would be collected in an anonymous database and managed in compliance with European data protection regulations and in accordance with the principles of the Declaration of Helsinki (40). The final protocol was approved by the Ethics Committee of the Azienda Ospedaliero-Universitaria di Cagliari, Italy. The trial was registered on ClinicalTrials.gov with the registration number NCT03858114 and approved by the Independent Ethics Committee of the University Hospital of Cagliari with approval number PG/2018/15546.

## Results

As shown in **Table 1**, the study sample has an average age of 73.36 ± 4.97, and it is relatively well balanced by sex (53.8% women). The samples extracted from two community surveys are balanced by age and sex. In the sample study, the pre-post pandemic comparison in the frequencies of outcome measures is shown in **Table 2**. The mean SF-12 score did not show statistical differences; only a slight tendency to decrease in the lockdown period (pre: 35.73 ± 4.52; post: 34.32 ± 6.42; one-way ANOVA 1.84 df; F=2.999; p=0.085). The mean PHQ-9 score was found to be homogeneous in both observation times (pre: 2.25 ± 3.04; post: 2.34 ± 3.49; one-way ANOVA 1.84 df; F=0.035; p=0.851). The frequency of depressive episodes before and during the pandemic, measured with the PHQ-9 cut-off 9/10 (i.e., including only severe depressive episodes) was not statistically different. Although, there was a slight tendency in its increase during the lockdown (3.22% vs 6.45%; chi-square 1df =1.051; p=0.305). Moreover, the frequency of depressive episodes measured with PHQ-9 cut-off 4/5 (i.e., including depressive episodes even of mild severity) was homogeneous. The analysis does not detect pre-post differences in the sub-groups divided by sex and age using 74/75 years of age as a cut-off (**Table 2**). The study sample at T1 showed an average SF-12 score that doesn’t differ from the sample obtained by the Italian population community sample (34.32 ± 6.42 vs 35.48 ± 7.02; one-way ANOVA 1,277 df; F=1.790; p=0.182). Otherwise, the frequency of depressive episodes doesn’t differ from the sample extracted from a Sardinian community sample (6.45% vs 10.75%; Chi-square 1df =1.357; p=0.244) (**Table 3**).

**Table 1:** Demographic characteristics of older adult samples during COVID-19 and control groups matched 2/1 by sex and age.

Socio-demographic	Older adults sample during COVID-19 pandemic N=93	National survey on H-QoL (25) N=186	Regional survey “loss of sadness” (26) N=186
Females (%)	50 [53.8%]	100 [53.8%]	100 [53.8%]
		(χ2 = 0.000, p = 1)	χ2 = 0.000, p = 1)
Age	73.36 ± 4.97	73.36 ± 4.96	73.48 ± 5.11
		One-way ANOVA 1,277 df; F=0,000; p=1	One-way ANOVA 1,277 df; F=0,035; p=852

**Table 2:** Pre-post pandemic comparison in the frequencies of outcome measures in the study sample.

Scales	Older adults sample before COVID-19 N=93 (T0)	Older adults sample during COVID-19 N=93 (T1)	T0 vs. T1 comparison
SF-12 Mean Score	35.73 ± 4.52	34.32 ± 6.42	One-way ANOVA 1,84 df; F=2.999; p=0.085
PHQ-9 Mean Score	2.25 ± 3.04	2.34 ± 3.49	One-way ANOVA 1,84 df; F=0.035; p=0.851
PHQ-9 > 9	3 (3.22%)	6 (6.45%)	Chi-square 1df =1.051; p=0.305
PHQ-9 > 4	16 (17.20%)	15 (16.2%)	Chi-square 1df =1.051; p=0.305
PHQ-9 > 9 in males	0/43 [%]	1/43 [2.33%]	Fisher Exact Test according to time and group (pre/post: M/F)
PHQ/9 > 9 in females	3/50 [6%]	5/50 [10%]	Male vs. female p=0.999
PHQ-9 > 4 in males	5/43 [11.6%]	4/43 [9.3%]	Fisher Exact Test according to time & group (pre/post: M/F)
PHQ-9 > 4 in females	11/50 [22%]	11/50 [22%]	Male vs. female p=0.999
<75 years old PHQ-9 > 9	2/57 [3.5%]	3/57 [5.3%]	Fisher Exact Test according to time &group (pre/post: <75/>74F)
>74 years old PHQ-9 > 9	1/36 [2.8%]	3/36 [8.3%]	<75 vs. >74 p=0.999
<75 years old PHQ-9 > 4	9/57 [15.8%]	7/57 [12.3%]	Fisher Exact Test according to time &group (pre/post: <75/>74F)
>74 years old PHQ-9>4	7/36 [19.4%]	8/36 [22.2%]	<75 vs. >74 p=0.724

**Table 3:** Comparison of the frequencies of outcome measures in the study samples.

Scales	Older adult samples during COVID-19 N=93	National survey on H-QoL (27) N=186	Regional survey “Loss of Sadness” (28) N=186	Statistics
SF-12 Mean Score	34.32 ± 6.42	35.48 ± 7.02	————	One-way ANOVA 1,277 df; F=1.790; p=0.182
PHQ-9>9	6 (6.45%)	—–	20 (10.75%)	Chi-square 1df =1.357; p=0.244

## Discussion

Our study found that a sample of older adults from the Sardinia region, Italy, did not significantly worsen their perception of H-QoL during the COVID-19 lockdown comparing with measured one year before. Also, the frequency of depressive disorders identified through PHQ-9 did not worsen. Neither the level of perception of H-QoL nor the frequency of depressive episodes worsen during the lockdown, as evidenced by large samples from large community samples. These results shown a remarkable resilience found in older adults during the pandemic, despite the extreme life-threatening condition amplified by the media with discouraging data on mortality (41). Our study confirms the results of other surveys in western countries that have detected low rates of stress and depressive disorders in the older people during the pandemic and the lockdown (7, 8, 42), but not in other cultural settings (43). However, these results seem even more significant if we consider that in those months Italy had among the highest mortality rates in the world (44). Therefore, might be useful to discuss which factors may have influenced the resilience in older adults (45). The American Psychological Association defines resilience as a “process of adapting well in the face of adversity, trauma, tragedy, threats, or even significant sources of stress” (46). This definition, although meaningful, may not include all the multifaceted components and the complex nature of this process nor its potential variability across different cultural, economic, and social settings (47, 48) Community networks may have played a protective role in Italy, specifically informal networks of support for the elderly (49). In southern Italy, the family still includes an extended circle and a network with constant support to family members through phone calls and social networks (49, 50). In fact, Sardinia maintains a strong family tradition where the municipalities delivered a home distribution of food and medicines to the older adults avoiding exposure to the infective risk (51). Moreover, it was shown that better quality of life in older people was associated with better social support systems across different EU countries (52). The absence of statistically significant differences in depressive symptoms and health-related quality of life between the pre-lockdown and lockdown assessments should be interpreted in light of several factors. First, the study’s modest sample size, while sufficient for detecting moderate-to-large effects, may have limited the statistical power to identify smaller yet clinically meaningful changes. Nevertheless, the use of matched control samples from large epidemiological datasets partially mitigates this limitation by enhancing the robustness of comparisons. Second, the baseline characteristics of the cohort, comprising relatively healthy, socially engaged, and motivated older adults, may have contributed to a ceiling effect in both depression and quality of life scores, reducing the likelihood of substantial deterioration over time. Third, the sociocultural context of Sardinia, characterized by strong family networks, intergenerational support, and stable daily rhythms, may have acted as a buffer against the psychological impact of lockdown (51, 53). These findings are consistent with recent Italian data showing no significant increase in depressive symptoms among older adults during the first pandemic wave, despite widespread social restrictions (26). They also align with studies from other Western countries reporting stability or even improvement in mental health indicators among older adults during lockdown, a trend attributed to greater emotional regulation, reduced exposure to pandemic-related media stress, and adaptive coping strategies (12, 51). Conversely, evidence from other cultural contexts, such as East Asia, indicates higher vulnerability in older adults, potentially due to differences in household structure, digital connectivity, and access to community support during crises (43). Another synergic element of protection for this population could have been economic stability, although pension income is not high as in other European countries. A recent study conducted in a large community during the pandemic has shown that an important risk factor for depressive episodes was job loss or the presence of economic needs (54). Elderly individuals living in economically vulnerable regions were exposed to social isolation and experienced a significant decline in their quality of life, particularly between 2019 and 2022 (52, 53). Another factor that could play a relevant role is the stability of biological and social rhythms. Older adults seem to show a good reactivity to stress, a better emotional regulation and a better ability to maintain regular social rhythms rather than young people (55). The regularity of social rhythms is an important protective factor against mood disorders (26, 56). On the other hand, regularity of biological rhythms is linked to the light pollution that influences the secretion of melatonin and neurosteroids, known as a risk factor for mood disorders (57, 58). However, alterations of social rhythms could lead to a syndrome composed of an alteration in rhythms, stress and hyperactivity (DYMERS) (59–61). It must be considered that the lockdown, in addition to disrupting social rhythms, has increased the hours of dependence on e-pads and other internet connection devices (62). It dramatically increased light pollution related to device use and abuse (63). In the last years, over one-third of internet users worldwide were aged between 25 and 34 years, while those 65 or older represented less than 4.5% of all internet users (64, 65).

### Limitations

The inclusion criteria targeted elderly individuals living at home with chronic diseases, excluding only those with severe impairments or obesity, the latter being relatively uncommon among the Sardinian elderly population (66). However, participation in the active aging project was voluntary, which may have introduced a selection bias. The sample likely overrepresents individuals who are relatively healthy, motivated, and functionally independent, and who are more inclined to engage in social and health-related initiatives. Generally, hyperactive and novelty-seeking people could be self-selected; thus, people with a personality profile (67, 68)were supposed to be particularly resilient in social transformation or threat conditions (42, 59, 69, 70). As a result, individuals who are less motivated or unable to participate due to their physical or cognitive condition, often the ones who would benefit most from targeted interventions, are underrepresented in the sample.

## Conclusions

The stability of depressive symptoms and perceived quality of life observed in our cohort of older adults, despite the extreme social restrictions imposed during the lockdown, suggests the possible role of protective factors previously identified in this population, such as greater stability of biological and social rhythms (14) and reduced exposure to nocturnal light pollution from prolonged device use, both of which are known to support circadian rhythm regulation and mood stability in older adults (73). Although these variables were not directly measured in the present study, their relevance is supported by converging evidence from prior literature, warranting further investigation in future longitudinal research. The study highlights how a sample of older adults from Sardinia (Italy), does not show an increase in the frequency of depressive episodes or a worsening of the perceived quality of life during the lockdown. Furthermore, the frequency of these indicators does not differ compared to large samples extracted from community survey databases. The study suggests that a protective factors (greater stability of biological and social rhythms, less exposure to light pollution from internet devices) may have played a role, together with specific components linked to the economic and social conditions, not ignoring the traits of hyperactivity and seeking of novelties as particularity of this cohort.

## Data Availability

The datasets presented in this article are not readily available due to privacy and ethical issues. Requests to access the datasets should be directed to Viviana Forte, vivianaforte@gmail.com.
